# Barriers and Facilitators of Weight Management among School Children with Obesity: A Qualitative Investigation of Parents’ Perceptions

**DOI:** 10.3390/nu14235117

**Published:** 2022-12-01

**Authors:** Sara Zuarub, Lily Stojanovska, Habiba I. Ali

**Affiliations:** 1 Department of Nutrition and Health, College of Medicine and Health Sciences, United Arab Emirates University, Al Ain 15551, United Arab Emirates; 2 Institute of Health and Sport, Victoria University, Melbourne, VIC 3011, Australia

**Keywords:** school children, obesity, parent’s perception, healthy eating, weight management, barriers, facilitators, United Arab Emirates

## Abstract

Parents play a critical role in influencing the eating habits of their children. This study examined parents’ perceptions of factors that contributed to childhood obesity and sought their suggestions on various modalities for overcoming the barriers to healthy eating. Semi-structured in-depth interviews were undertaken with 26 parents of 9–13 years old school children with obesity from the United Arab Emirates. Three main topics covered in the interviews were: (1) Parents’ perceptions of their children’s weight and eating habits; (2) Attitudes towards healthy eating and weight management; and (3) Suggestions on how their children can adopt a healthy lifestyle. Interview transcripts were thematically analyzed using the NVIVO software to identify the emerging main themes and sub-themes. Parents identified individual/intrapersonal (child), interpersonal (peers, family, nannies), and institutional/school environment barriers and facilitators to a healthier lifestyle. The three major themes that emerged were: (1) Negative effects of obesity on children’s lives; (2) Barriers to weight management and healthy lifestyle; and (3) Facilitators to healthy eating. Nutritional education and a supportive home and school environment were suggested for the adoption of a healthy lifestyle by children.

## 1. Introduction

The prevalence of childhood and adolescent obesity remains a significant burden globally. Forty million children under the age of 5 and over 330 million children older than 5 years and adolescents have obesity [1]. The global challenge posed by obesity has prompted the World Health Organization to start a campaign called “no increase in childhood overweight by 2025” [2]. This initiative targets the prevention of various diseases associated with childhood and adolescent obesity. The short-term effects of obesity in school children include psychological conditions such as low self-esteem, anxiety, and depression [1,3,4]. Furthermore, the potential long-term effects include heart disease, type 2 diabetes, liver complications, cardiovascular diseases, and certain types of cancer [1,3,5]. The risks of these illnesses progress into adulthood and may cause musculoskeletal deformities and even premature mortality [1]. Therefore, curbing obesity prevalence is crucial in reducing the incidences of chronic illnesses in adulthood.

The Middle East is one of the regions with a high prevalence of adolescent obesity [5]. In the United Arab Emirates (UAE), an alarming upsurge in obesity has been observed in most schools [6]. In the last two decades, overweight and obesity cases have increased by two folds in adolescents aged 11 to 18 years [7]. The prevalence of obesity in the UAE has dramatically escalated to about 32.3% [8] Moreover, studies indicate that the annual increase in childhood obesity countrywide is 2.36% in children and adolescents aged 3 to 18 years [6]. Furthermore, several studies have reported a high prevalence of adolescent obesity in the UAE [6,7,9]. Bani-Issa et al. [9] revealed that the obesity prevalence in the UAE schools is 34.7%, with one of the Emirates, Ras Al-Khaimah public schools, students aged 11–18 years showed an obesity prevalence of 38–41.2% [6]. Similarly, Pengpid and Peltzer [7] suggested that in the pre-teenagers and teenagers aged group 8 to 14 years in the UAE schools, the obesity prevalence in males was considerably higher (42.1%) compared to females (35.6%). Therefore, it is crucial to develop effective strategies for reducing adolescent obesity in the UAE.

Parental control is crucial in promoting healthy eating and dietary practices in school children aged 9 to 13 years, as parents can heavily influence the eating habits of pre-teenagers and teenagers [10]. Parents’ knowledge and attitude towards healthy eating impact children’s food choices, meal patterns, and level of physical activity [11]. Therefore, parents have a high level of influence on the children’s weight and nutritional status [10,11]. Moreover, studies suggest that including parents in the prevention strategies can substantially improve the weight of their obese children [10,11]. Although there is a consensus globally that prevention is a crucial strategy for reducing the obesity rate, limited success has been achieved so far in implementing this approach in school children alone. Parents play a critical role in influencing the dietary habits of children in their early years [11,12]. Previous research identified the obesity risk factors in children to include high consumption of fast foods and sugar, low intake of fruit and vegetable, sedentary lifestyle, as well as eating large portions of foods [13]. However, suitable strategies for supporting parents in improving their children’s nutritional status still need to be identified. Nonetheless, seeking parents’ opinions on how to promote healthy eating and exploring their attitudes toward their children’s eating behavior and physical activity is crucial in developing effective prevention strategies [14].

According to the Social Ecological Model (SEM) [15], these factors are classified into intrapersonal, interpersonal, institutional, and environmental [15]. Physical activity (PA) also plays a key role in managing the weight and well-being of the children. It has been shown that PA improves the development of not only the physical characteristics of the body but also the mental capacities in school-age children [16]. However, at present there are specific barriers to engage in an adequate level of physical activity among schoolchildren, including lack of time, desire, skills, resources, along with social and cultural influences [17]. However, increasing burdens on children, due to modernization of education, and the transition to technological capabilities, together with global challenges like a pandemic, create an environment that is not conducive to PA, even in the absence of apparent restrictions [18]. The UAE is equally susceptible to these factors, which require a modification of some lifestyle habits and behaviors at the level of school children. This problem is especially relevant since school-age children require an appropriate level of PA for healthy growth.

This study explored the views of parents on factors that contributed to childhood obesity and sought their suggestions on possible ways of overcoming barriers to weight management and the adoption of healthy eating. This requires a qualitative approach to evaluate the current trends and beliefs among the UAE- families associated with childhood obesity, as well as strategies to prevent this. There is a lack of studies seeking parents’ opinions on factors that increase the risk of being overweight and obesity among school children in the UAE. Using the results obtained from this study, could assist in developing targeted, evidence-based interventions that can facilitate healthy nutritional strategies for families with overweight and obese children in this country.

## 2. Materials and Methods

### 2.1. Theoretical Framework

The increased rise in the global incidence of childhood obesity has now been identified as a severe public health problem [19]. Failure to identify and comprehend the environmental and internal elements that contribute to successful weight reduction may lead to the inefficacy of existing treatment approaches [20]. Many factors can affect school-age children that contributes to obesity. This study applied the Social Ecological Model (SEM) [15] to explore the multiple factors that can contribute to obesity among school children from the perspectives of parents. The multiple levels of the SEM (interpersonal, interpersonal, community, organizational, policy) provide a useful framework for understanding the multiple factors that influence the adoption of healthy lifestyles among school children, including individual preferences, families, peers, and school environment) [15,20].

Concerning school-age children, the influence of such determinants is especially significant since it falls at the child’s rapid development stage. In this situation, there are many stakeholders, including the children and their parents, teachers, friends, relatives, and other society representatives with whom the students communicate. Each of the parties contributes to the formation of the child’s lifestyle and brings in those factors that contribute to or hinder obesity to varying degrees.

Parents are the closest people to each child and know much more about their characteristics than other stakeholders. This issue primarily concerns the health of schoolchildren, but knowledge of other psychological or behavioral traits can contribute to effective intervention in treatment. The level of the SEM relationship model is closely related to the individual in the context of schoolchildren. Children at this age are emerging personalities, and, as a rule, their parents influence them most significantly. At the same time, schoolchildren are open to new influences from outside, making each case unique, and presenting a person as a complex and super-dynamic system. In this regard, parents’ opinions should be sought since they may have the most relevant information necessary to correctly understand the factors influencing their children’s adoption of healthy lifestyles.

### 2.2. Study Design

This is a qualitative study involving in-depth interviews with parents of 9–13-year-old children with obesity who attended government schools in the two Emirates in the UAE, Sharjah, and Dubai. A qualitative research method was deemed to be the most appropriate in studying parents’ perspectives on childhood obesity (opinions, experiences, and suggestions), allowing themes to emerge. The parents were invited to express their views on factors that contributed to childhood obesity and sought their suggestions on possible ways of overcoming the barriers to weight management and the adoption of healthy eating among school children. The study was conducted to assist in the development of a planned intervention for overweight and obese 9–13-year-old school-aged children in Dubai and Sharjah. Data were collected from September to November 2021.

### 2.3. Participant Recruitment

Parents of school children who were overweight or obese were invited to participate in individual interviews. The inclusion criteria were: Living in the Emirate of Dubai or Sharjah in the UAE and having a child aged 9–13 years with overweight or obese attending one of the 36 schools selected for a planned nutrition education intervention. Parents of children who were not overweight or obese and those whose children were not attending the schools selected for the planned intervention were excluded.

The purposive sampling technique was used to recruit parents of 9–13 school children with obesity attending public schools in the above-mentioned two Emirates for an individual interview. Purpose sampling allows the selection of participants based on predetermined criteria relevant to a particular research question to obtain rich data [21]. The basis of sample size determination was data saturation. Parents of overweight/obese 9–13-year old children participating in a school-based intervention were contacted through emails explaining the purpose of the study and the main topics to be discussed during the interview. They were invited to attend a single in-depth individual interview. Those interested were asked to confirm their willingness to participate in the study by responding to the email and choosing their available date and time for the interview. Of those contacted, 35 parents accepted to participate in the interviews.

### 2.4. Semi-Structured Interviews

At the beginning of each interview, the facilitator collected information about the participants’ gender, age, nationality, employment, and marital status. A semi-structured interview guide (Supplementary Materials Table S1) was developed as a guide during the interview process. It contained 13 key questions focusing on three main areas: (1) the perception of the parents regarding their children’s weight and eating habits, (2) their attitude towards healthy eating and weight management through nutritional interventions, and (3) their suggestions on how the parents and the school administration can support the children in adopting healthy eating pattern and effective weight management. The research team consisting of a nutritionist and a university faculty member developed the 13 questions guided by the related literature [22,23,24,25]. Before initiating data collection, the interview guide was translated into Arabic and subjected to content validation [26]. This was subsequently pilot-tested with 6 parents of children aged 9–13 years with obesity (three females and three males). Necessary modifications to the questions before the commencement of the study were made accordingly. In-depth semi-structured individual interviews were conducted with parents of 9–13-year-old school children who were overweight or obese. The interviews were conducted in Arabic virtually, which was their preference. The interview duration ranged between 45–60 min, including the time used to explain the purpose of the interview. Additional probing questions were used to seek further information or clarifications when needed. All the interviews were collected by a female native Arabic-speaking senior nutritionist trained in qualitative research data collection techniques who was a doctoral student in nutrition and was not affiliated with any of the schools where the children of the parents were attending. The interviewer had no previous contact with the participants except by sending an invitation email for the interviews. All discussions were audio-recorded. At the beginning of each interview, the interviewer obtained the participant’s verbal consent, explaining the purpose of the study, and assuring the participant of the confidentiality of the discussions. The participants were also informed that they have the right not to answer any question they do not wish to answer or discontinue the interview at any time. In addition, they were assured that all the information collected would remain confidential and would be used only to meet the purpose of the study. Throughout the data collection, the interviewer reflected on the interview guide and on her attitude during interviews to reduce interviewer bias [27]. The recordings were listened to as the interviews progressed to identify topics to explore in subsequent interviews. After 26 interviews, no new information was provided by the subsequent interviews that could contribute to the study findings, indicating that data saturation was achieved [28,29]; thus, the process of data collection was concluded.

### 2.5. Data Analysis

The interview recordings were used to transcribe the discussions verbatim in Arabic. The transcripts were translated into English by the interviewer so that the text-based analysis could be performed using the NVIVO software (NVIVO, 12, QSR International). Translated interview transcripts were imported into NVIVO 12 to conduct the analysis and facilitate the development of the codes using an inductive thematic approach [30]. As the interviews progressed, data were analyzed (i.e., concurrent data collection and analysis) to identify initial codes and categories. The interview questions were refined with additional probes based on this preliminary analysis in line with the qualitative research methodology [31]. The constant comparison method [32] was used in identifying recurring data, creating categories, systematically comparing and grouping them into themes, The transcripts were read multiple times to familiarize with the data and to identify initial codes. The codes were constantly compared and then grouped into categories/subthemes, which were later also grouped into themes [30]. The coding was performed by the first author (SZ), and the coding assignment and appropriateness of the codes to the assigned themes and sub-themes were reviewed by the third author (HIA), who has an extensive background in qualitative research. Any inconsistencies were resolved through discussion to reach a consensus.

### 2.6. Quality Assurance

Multiple approaches were employed to enhance the quality of the study. The equivalency of the Arabic transcriptions and the translations of Arabic into English were checked by two independent reviewers fluent in both Arabic and English. In addition, 15 of the recorded interviews were sent to an external reviewer along with the translated transcript to check the consistency of the English translation against the Arabic recordings. Finally, the translated summary of the interviews was sent to six parents who were fluent in English to confirm the accuracy of the translated text with the discussions during their interviews. Participant quotes that support the emerged themes and sub-themes are presented in the Results section. The interviews were recorded and transcribed verbatim in Arabic, and participant quotes are provided in the Results to enhance credibility. The interview transcripts were shared with the participants to confirm their accuracy, and the English translation of the Arabic transcripts and the transcription of the Arabic from the recordings underwent independent reviews. Finally, we have provided COREQ (COnsolidated criteria for REporting Qualitative research) Checklist [33] as Supplementary Materials Table S2.

### 2.7. Ethical Approval

All the participants of the study were fully informed of its objectives, purpose, and methods used before they gave their consent to participate Moreover, the researchers avoided revealing any names or other specific details to protect the identity of the participants and maintain confidentiality. The ethical approval for the study was obtained from the United Arab Emirates University Social Sciences Ethics (protocol number: ERSC_2022_744). All procedures followed the principles of the Declaration of Helsinki.

## 3. Results

A total of 26 parents participated in the individual interviews, with the majority (88%) being females. All interviewed parents were Emiratis, and the vast majority were working (92%). Table 1 below presents the demographic information of interviewed parents.

Results of the thematic analysis of conducted in-depth interview transcripts identified 3 main themes: (1) Negative effects of obesity on children’s lives; (2) Barriers to weight management and healthy eating; and (3) Facilitators to healthy eating. There were 8 subthemes related to the 3 major themes. The themes were categorized into three groups, namely Individual/intrapersonal (child), interpersonal (peers, family, nannies, etc.), and institutional/school environment).

### 3.1. Theme 1: Negative Effect of Obesity on Children’s Lives

Being overweight has various adverse effects on children’s lives at the physical and psycho-social levels, as mentioned by interviewed parents. The parents were informed that their children’s weight affects their ability to play with friends. One of the interviewees, a male father of an overweight child, stated:

*“My child’s weight is greatly affecting his life. He wishes he could run with his friends and ride a bike, but he can’t because of his weight.”*

#### 3.1.1. Individual/Intrapersonal Issues

**Negative effect on self-esteem**: Parents indicated that obesity results in social isolation of children, which can be attributed to an intrapersonal barrier of low self-esteem. Due to the presence of stereotypes concerning weight, children with obesity tend to develop self-esteem issues, thus, choosing to abstain from communicating with others. It leads to limited interactions with peers, which negatively impacts the child’s development of social skills and the adoption of healthy eating. The mother of one of the children commented:

*“His weight stops him from playing with other kids, which means his social skills will not develop over time; and for sure, this will affect his psychological well-being. Therefore, he will not be motivated to achieve his goals.”*

#### 3.1.2. Interpersonal Issues

**Social isolation and Poor social skills**: Social isolation of overweight and obese children can be caused by bullying from peers as another woman points out:

*“He is being bullied at school and among his friends because of his extra weight, and this is what makes him hate going to school and hates studying and playing with his friends.”*

Some parents indicated that their children’s weight is not affecting their lives currently. However, they expressed concern about their prospects in the future and how further development of children’s social skills might be limited by obesity:

*“While my child is a little bit overweight for his age, I believe his weight does not stop him from achieving his goals at the moment, but in the future, it may become a major limitation.” Therefore, peer pressure and bullying can be seen as significant interpersonal barriers.”*

#### 3.1.3. Institutional/School Environment Issues

**Bullying by schoolmates**: A lack of support from classmates addressing the needs of children with obesity issues ought to be considered. Indeed, cases of bullying and harassment are becoming more common as children grow and begin to compete for their status in class or other school groups. One mother noted,

*“My son is constantly insulted by his classmates who call him a lame duck and a loser. The bullying intensifies after physical education classes when other school children make jokes about his inability to do some exercises.”*

**Poor academic performance and lack of interest in school**: These two can also contribute to weight gain. Indeed, bad marks serve as stress factors that may trigger overeating to suppress anxiety. Three parents noted that getting bad marks at school made their children eat more at home to curb the feeling of failure:

*“I have noticed that when my son gets bad marks or lower grades than usual he starts to eat more and more without stopping, he is like running from the stress and disappointment towards food, especially sugary snacks.”*

Lack of interest in school may also serve as a trigger for weight gain. Indeed, two parents noted that their children have no interest in classes and have snacks to fill in the time. One mother said:

*“My daughter complains of being bored at school with no interesting subjects or teachers who can inspire her in her studies. She claims that the dining room during the breaks is the only place she gladly visits since the snacks they offer there are delicious.”*

### 3.2. Theme 2: Barriers to Healthy Eating

During the interviews, parents discussed and highlighted various perceived barriers hindering their children from eating healthy, which eventually caused them unwanted weight gain. Barriers are described in this study as factors that make weight loss or sustaining healthy eating difficult for children aged 9–13 years. The discussed barriers were classified into three categories: Individual/intrapersonal (child), interpersonal (peers, family, nannies), and institutional/School environment).

#### 3.2.1. Individual/intrapersonal Barriers

In this study, individual/intrapersonal barriers are considered to be existing within the child. These barriers were reflected in the following sub-themes: (1) Sedentary lifestyle, (2) Personal food choices, and (3) Low nutrition knowledge.

**Lack of physical activity due to a sedentary lifestyle**: With the emergence of COVID-19, children were bound to stay and study from home for extended periods. It also drastically reduced their time outside their homes and their activities with friends, hence, reducing their physical activity. As one of the mothers commented on her son’s behavior:

*“His weight is constantly increasing.” He is less mobile due to sitting in front of the computer for extended periods because of remote education.”*

In addition, parents discussed their concerns about their children’s preference to stay at home and engage in social media and video games:

*“I believe that the main reason my child is overweight is that he does not move enough, and his only activities involve watching T.V. and sitting in front of the computer.”*

**Personal food choices**: Children’s strong interest in purchasing junk food and their preference for certain food products over others also influences their eating habits. Parents reported that their children prefer fast foods over more healthy dishes. Moreover, some said that their children are not willing to decrease their consumption of unhealthy food:

*“His love for food that is full of calories, flavors, and harmful juices is the main issue. Most of the time, he refuses to eat healthy snacks.”*

The other personal barrier parents revealed was the unwillingness of their children to reduce the consumption of unhealthy snacking. One of the parents pointed out that their children excessively love breakfast consisting of junk food:

*“He loves breakfast with biscuits and chips and loves to eat noodles and pastries daily.”*

**Low nutritional knowledge**: Lack of a credible source continuously disseminating nutrition and food-related information hinders establishing healthy nutrition practices. Parents mentioned their need for credible sources of nutrition information:

*“My son’s lack of knowledge on how certain foods have to be consumed in moderation is the main problem that causes him to have such significant portions of snacks that he consumes almost daily. More accessible knowledge about healthy eating could help to encourage children to eat more healthy food.”*

#### 3.2.2. Interpersonal Barriers

For this study, interpersonal (peers, family, nannies) barriers are considered to be the factors that stem outside the child, which include a relationship with parents, family, nannies, and others. These external barriers were reflected in the following sub-themes: (1) peer pressure and perceived negative influence of peers and social media, (2) limited parental engagement and parent’s role in children’s lives, and (3) unsupportive nannies.

**Peer pressure**: The parents identified peers as the other critical areas of concern. One of the parents revealed that some children might tend to eat unhealthy foods when they are in the company of their friends:

*“My son often drinks sodas when he is with friends.”*

Moreover, the option of controlling what their children eat in the company of their friends elicited mixed reactions. Some of them admitted talking to the parents of the other children to discourage unhealthy habits, whereas some were reluctant. Some parents also revealed that healthy traditional meals are rarely showcased on social media compared to unhealthy ones and attributed them to unhealthy eating habits. A mother of a slightly overweight child stated:

*“As I noted earlier, his sudden obsession with fast food is the main factor affecting his weight. Perhaps this new eating habit was influenced by social media and his schoolfellows.”*

**Limited parent engagement**: Parents’ preoccupation with their jobs and careers, especially mothers, negatively affected the children’s healthy eating habits and, in some cases, gave them a chance to eat whatever they wanted without proper monitoring. An illustration of this is the statement of the father of one of the children,

*“I think that the fact our son is almost fully responsible for what he eats, and we cannot monitor him, is the main factor why he cannot control his portion sizes and the types of food products he consumes.”*

Moreover, majority of the parents mentioned that their busy and demanding work schedule results in their inability to cook healthy food, feed their children healthy food, or monitor their children’s eating habits and food portions. The mother of one of the children noted:

*“Since I am a working mother, I do not have much time to cook healthy meals daily. Most of our meals consist of frozen food or food filled with starchy foods such as rice and pasta, in addition to our heavy reliance on takeaway.”*

Parents’ unhealthy eating habits were also identified among the underlying factors of children’s unhealthy eating patterns. One mother commented on her son’s behavior:

*“During the weekends, we often go out to fast-food restaurants or order food at home, so he sees me engaging in similarly bad dietary behavior. The fact that his dad and I have never been preoccupied with healthy eating has negatively influenced my child’s own eating preferences and led to his weight gain.”*

In addition, parents identified that buying their children sweets and sweetened juices and keeping these products at home is also one of the internal barriers hindering their children’s ability to have healthy eating habits:

*“I often share fast food with him and buy him, sweets, if he asks for them.”*

**Unsupportive nannies**: The involvement of the nannies in the children’s everyday life affects negatively their healthy eating. According to some mothers, the nanny might be less strict with the children than the parents are:

*“…The nanny usually gives him anything to keep him quiet and safe, so he picked this habit of having whatever he desires without any restrictions. This behavior allows nannies to keep the child in a positive mood but significantly affects their eating habits.”*

#### 3.2.3. Institutional/School Environment Barriers

This study defines institutional/school environment barriers as factors that originate within the school that limits healthy eating or healthy weight maintenance among school children aged 8–14 years old. The subthemes identified for this category include (1) Lack of nutritional education in schools and (2) School demands that lead to stressful eating and lack of time for physical activity.

**Lack of nutritional education in schools**: Most parents noted that their children are not taught nutritional education at school. The school curriculum needs to specifically focus on the need for healthy eating habits and how to achieve them. Parents cited that most of the children are not able to use the knowledge acquired at school in general science classes as they do not relate to their daily lives. As stated by one parent:

*“My son knows different food categories and their functions but still cannot use them to control his food portion. The knowledge he has is confusing for him”*

School demands lead to stressful eating and a lack of time for physical activity:

The busy schedule in school and the demand that school has on children create anxiety for most students. One of the parents said:

*“My daughter, instead of losing weight, gains more weight due to the stress of schoolwork. She tends to eat a lot of snacks when she comes from school and does not control her portions.”*

Another parent noted that her son comes home with a lot of homework, and he does not find time for physical activity or exercise,

*“Nowadays, teachers demand more from kids, and the amount of homework is mind-blowing. My kid goes to school to study, then comes back to study even more. He does not have any time for exercise.”*

### 3.3. Theme 3: Facilitators for a Healthy Eating

Throughout the interviews, parents mentioned and discussed various perceived facilitators (enablers) that might positively affect their children’s attitudes and behaviors toward healthy eating. These facilitators are presented in three categories: intrapersonal, interpersonal, and institutional facilitators.

#### 3.3.1. Intrapersonal Facilitators

For this study, intrapersonal facilitators are considered as all the factors that children can do to optimize their healthy eating. These facilitators were reflected in the following sub-themes: (1) Motivation to engage in physical exercise and (2) Healthy eating behaviors.

**Motivation to engage in physical exercise**: Intrinsic motivation is an essential facilitator of healthy eating. Parents have reported that the children lose more weight when they are self-driven than when being pushed. Taking personal responsibility for their health makes the children more engaged in physical activity. Additionally, with motivation, they are not easily discouraged when things are complicated, and the goal is not achieved instantly. As reported by one parent, her son had shown significant improvement when he was enthusiastic about his physical activity.

**Healthy eating behaviors**: Healthy eating habits are identified as one of the facilitators of healthy eating. As reported by parents, healthy eating habits include eating patterns such as controlled food portions and food substitution. One parent indicated that substituting healthy snacks like fruits and vegetables for snacks with refined sugar is essential in the weight loss and control journey:

*“Sugar is essential for young children, yet candy with harmful ingredients can easily be replaced with fruits or foods cooked with organic ingredients.”*

The parents noted that despite being hard for children to practice healthy eating behavior, once they recognize the importance, they can stick to it. According to the parents:

*“The children can learn the importance of healthy eating by watching documentaries or learning how junk food is made.”*

#### 3.3.2. Interpersonal Facilitators

In this study, interpersonal facilitators are considered as all the factors that encourage and optimize children’s healthy eating that is outside the control of the children. These interpersonal facilitators were reflected in the following sub-themes: (1) Parents’ healthy eating perception, (2) Parents’ positive attitude, and (3) Advise from a nutritionist.

**Parents’ perception of healthy eating**: Parents discussed their knowledge about the benefits of following healthy eating and highlighted their perception of the importance of adopting this behavior for their children’s health in the future, as adults. One of the mothers stated:

*“Nutritional and health fields are critical as they are responsible for our psychological and nutritional health. If our food is healthy, we will be in the best physical and psychological health.”*

**Parents’ positive attitude**: The parents expressed their positive attitude toward being involved with their children on their journey toward healthy eating. They mentioned being ready to change their eating habits to influence their children to change their unhealthy eating habits positively. One mother also expressed their willingness to cook healthy food weekly for their children:

*“I can adopt a new healthy food plan by encouraging my son to eat food full of healthy vegetables and vitamins by buying an air fryer to cook with.”*

In addition, parents mentioned that they are ready to engage with their children in conversations about healthy food and the importance of following healthy eating habits, as noted by one of the parents:

*“The health of my child is essential to me, and if it takes a great effort to help them understand the importance of healthy food, I am ready to engage in this process.”*

They further mentioned establishing a reward system for their children whenever they eat healthy foods:

*“I can support my child by making healthy meals for him consisting of fruits and vegetables, beautifully decorating them, and giving him material rewards when he eats healthy food.”*

Furthermore, parents expressed readiness to stop buying their children unhealthy snacks and bring home more fruits and vegetables. One mother said:

*“I am a working mom who, unfortunately, does not have much free time. For a long time, I chose the meal that could be prepared in under an hour. However, now I realize how unhealthy this food is, which made me consider buying more fruits and vegetables.”*

**Advice from a nutritionist**: Parents highlighted that receiving advice from a **nutritionist** would significantly impact their and their children’s eating habits and encourage them to have healthy eating habits. One of the fathers stated:

*“Having a nutritionist’s advice will contribute to educating my child about the importance of eating healthy and the way to build a balanced meal plan.”*

#### 3.3.3. Institutional Facilitators

**Active school involvement**: The parents suggested several ways the school management can follow to support the children’s journey toward healthy eating habits. One of the mothers suggested raising awareness, by saying that:

*“The school administration can participate by publishing awareness campaigns in schools to reinforce the conviction of children about the importance of healthy meals for their physical health.”*

Another suggestion included the provision of nutrition information in the school curriculum:

*“The school administration can add study materials and courses of a nutrition curriculum that will be designed for school students, teaching them about healthy food and encouraging them to eat it, especially from young ages such as kindergarten students. Thus, the child will grow up healthy from childhood.”*

## 4. Discussion

The issue of childhood obesity in the UAE has reached an alarming rate [7,9], acting as a public health threat to children and adolescents. For this qualitative research, in-depth individual interviews were used to explore parents’ perceptions of factors that contribute to childhood obesity and their suggestions for healthy eating patterns in the prevention and management of overweight and obesity among school children. This method was considered the most appropriate in exploring and determining the attitude of parents towards healthy eating and their perception of their children’s weight and nutrition-related habits. In addition, this method can facilitate the parents’ recommendations and suggestions on ways the parents, the school administration, and professionals can support school children in adopting healthy eating habits.

**Figure 1:**
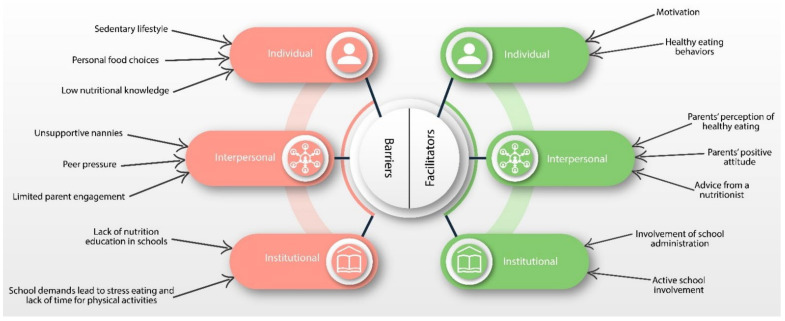
Barriers and Facilitators of health body weight among 9–13 old school children.

Overall, the results of our study indicate that childhood obesity continues to be a significant public health issue. Furthermore, the themes identified during the analysis indicate the necessity to involve parents actively in shaping the approach toward building healthier eating habits for their children. Unique nutritional requirements for each child must be integrated into the general nutritional plan for the school, therefore, providing the schools the opportunity to manage the issues directly. The results from our study have led to the development of a conceptual framework (Figure 1) based on the SEM [15]. This framework focuses on three critical elements of the SEM (individual level, interpersonal level, and institutional/organizational level). The conceptual framework was continuously updated and expanded throughout the study based on the findings to better reflect emerging themes and sub-themes and their interrelationships.

The parents identified various factors that can affect school children’s adoption of healthy eating and regular physical activity (healthy lifestyle). Some of these factors were barriers to the school children’s ability to adopt healthy eating, and others were facilitators enabling school children towards healthier eating styles. These factors act at the intrapersonal, interpersonal, and institutional levels. The adverse effects identified as barriers on the above-named levels drive school children towards adopting unhealthy behavior and becoming overweight.

Parents appeared eager to attempt new techniques to help their children reduce weight. The overarching issue was that parents were unable to find sufficient time to engage with their children due to working hours or other responsibilities. Parents stated that while purchasing healthy meals is an option, eating well is also more expensive. Given their busy routines, several parents said that fast food outlets were less expensive and even more convenient for feeding their children on occasion. The findings were corroborated by Vittrup and McClure [34], who revealed that the critical barrier to childhood obesity weight management is parents’ time deficiency in being directly involved. Importantly, Garza [35] examined nannies’ perception of children’s eating habits. The researchers recommended the provision of nutritional education for nannies to improve the children’s nutritional status.

Cultural differences caused by the characteristics and customs of different ethnicities are similar in the causes leading to obesity but differ in intensity. Among the children in the US., obesity rates are higher among the non-whites compared to the Caucasians [36]. One of the key determinants lie in the social status of children which often dictates place of residence (e.g., low-income vs. affluent neighborhoods) [36]. Extrapolating to the UAE, a previous study reported that traditional and cultural factors and ethnic differences can play a role in the risk of childhood obesity [37]. The present study is limited by geographical and cultural factors since the parents who participated in the interviews were all UAE nationals and the region of the study was limited to only two of the 7 emirates of the UAE. However, although income and place of residence have been associated with obesity [36], in the present study the role of the above two factors on the perspectives of the parents is expected to be shaped by the high standard of living Emirati nationals have.

The participants discussed the negative influence of peers and social media, common nutritional knowledge, and a sedentary lifestyle as some of the barriers. Our findings were consistent with Ragelienė and Grønhøj [38], who reported that family, peers, and social media influence children’s eating behavior. Therefore, positive role modeling from parents, nannies, and peers has the potential to promote healthy eating behaviors in children. Parents further indicated that other facilitators could play a significant role in helping their children adopt a healthier lifestyle and overcome being overweight. These factors were related to parents’ engagement and external factors.

Creating parental concern through knowledge of obesity health risks was identified as a critical factor in controlling childhood obesity [39]. Moore and colleagues [40] found that concerned parents were more likely to instill the desire to adopt healthy eating in their children, compared to the parents who paid no particular attention to forming healthy eating habits which is consistent with the findings of the present study. Aljunaibi et al. [11] evaluated parental perceptions of school children in the UAE and found that 63.5% of the parents of overweight/obese children misclassified their children’s body weight status. The study suggested parents who recognize the challenges associated with childhood obesity will actively participate in managing the weight of their children. These findings are consistent with Etelson et al. [39], who reported that parents of overweight children mostly had inaccurate perceptions regarding their children’s weight. Therefore, creating more awareness of the potential adverse effects of obesity on health and well-being would contribute to the reduction in childhood obesity in the UAE.

Overall, the suggestions provided by the parents were insightful and consistent with those previously reported in other studies [41,42]. El-Sabban [41] suggested that children’s nutrition and general health require the support of parents, teachers, school administrators, nannies, and other directly or indirectly involved parties. Hinojosa et al. [42] revealed that modification of the school environment is crucial in childhood obesity weight management. The institutions can incorporate more programs that involve nutritional education and physical activities. Additionally, surveillance programs can be initiated in public schools to identify the students who are obese. Subsequently, they can be supported through various nutritional interventions.

The barriers to healthy eating identified by parents can be addressed through the recommendations, including but not limited to: (1) Collaborative programs of nutritional education targeting the parents and children that can be developed by relevant government agencies to support the children in adopting healthy eating; (2) Implementing training programs for nannies to enhance their nutritional knowledge. They can be trained on how to support the children in adopting a healthy lifestyle, especially in instances where the parents are busy at work. This can be achieved through collaborative efforts between relevant government institutions that can improve access to nutritionists for families, including the nannies. Additionally, awareness campaigns can be initiated through television commercials and social media to counter the negative influence.

Furthermore, there is a need to implement more opportunities for children to be physically active at school. Additionally, surveillance programs to identify children at risk for obesity and intervene at an earlier stage must be developed. Addressing the negative peer influence from the interpersonal category by implementing nutritional awareness programs in communities and the formation of social support groups to help school children with obesity is vital. School nutrition policies play a key role in improving children’s nutritional health. Although, multiple authorities in the UAE developed and implemented various initiatives and guidelines related to school nutrition, a recent study highlighted the need for coordinated efforts and policies for implementation to reduce the prevalence of childhood obesity [43].

The strengths of our study lie in the fact that this is the first study in the UAE on school children’s body weight management using the SEM [15]. Moreover, all the information used to determine the barriers and facilitators of weight gain in this age group is based on the views of parents of school children with overweight or obesity; thus, giving the findings of this study a specific relevance to the factors influencing the risk of overweight and obesity in 9–13 old school children. In addition, school children’s parents who took part in the interview did not receive any monetary compensation for their time. This fact minimizes the possibility of purposeful distortion of facts and makes the interviews more credible in what they disclose about school children’s eating habits. On the other hand, the use of a purposive sampling process limits the generalization of the results of the study to parents of school children with obesity in the UAE. Although these parents can provide insightful information on factors contributing to their children’s excess weight, selection bias may occur. Moreover, since only 26 parents were interviewed from 2 of the 7 Emirates in the UAE, further studies with a larger parental cohort and wider geographical population from other Emirates are needed.

## 5. Conclusions

Obesity, energy-dense foods, and a sedentary lifestyle among children remain one issue in modern society in the UAE. This study revealed that only the combined efforts of schools, parents, and children themselves would be conducive to the proper management of body weight in schoolchildren. Parental control, support from teachers and classmates, and personal determination by the child to lose weight remain the key drivers in successfully managing the issue of extra weight in school children. While this research has indicated the main factors contributing to weight gain, further research is required to develop specific programs for addressing obesity among school children at the national level.

## Figures and Tables

**Table 1:** Demographic information of interviewed parents.

Characteristics	n (%)
Gender	
Male	3 (12%)
Female	23 (88%)
Nationality	
Emiratis	26 (100%)
Other	0 (0%)
Employment Status	
Employed	24 (92%)
Unemployed	2 (8%)
Marital Status	
Married	24 (92%)
Divorced	2 (8%)

## Data Availability

The dataset used to prepare this analysis is available from the corresponding author.

## Supplementary Materials

The following supporting information can be downloaded at: https://www.mdpi.com/article/10.3390/nu14235117/s1, Table S1: Table Semi-structured Interview Guide; Table S2: COREQ Checklist.
