# Supplementary material for: Barriers and Facilitators of Weight Management among School Children with Obesity: A Qualitative Investigation of Parents’ Perceptions
Source: Nutrients. 2022 Dec 1;14(23):5117. doi: 10.3390/nu14235117 (PMC9739786; doi:10.3390/nu14235117)
Supplement: Supplementary file 1 [file nutrients-14-05117-s001.zip › nutrients-1910741-supplementary/Table S1 -Semi-structured Interview Guide.pdf]

Table S1. Semi-structured Interview Guide.

1. How would you describe your child's eating habits?
2. How do you feel about your child's weight?
3. What do you think contributed to your child's current weight gain?
4. What do you think about preventing your child from losing weight?
5. Do you feel that your child's weight is hindering him from achieving his goal?
6. Tell me more about how your feeding habits and attitude have affected your child's eating habits?
7. What eating patterns or dietary practices at home do you feel contributed to your child's weight gain?
8. What can you change about your child's eating habits at home?
9. What do you think that the school can support your child?
10. How do you feel that you can support your child in changing his eating habits?
11. What eating patterns would you like to change to help your child succeed in this task?
12. Do you think nutritional intervention is important to you and your baby? Why and how?
13. What barriers do you feel prevent your child from eating healthy food?
